# Carbon incorporation effects and reaction mechanism of FeOCl cathode materials for chloride ion batteries

**DOI:** 10.1038/srep19448

**Published:** 2016-01-18

**Authors:** Xiangyu Zhao, Qiang Li, Tingting Yu, Meng Yang, Karin Fink, Xiaodong Shen

**Affiliations:** 1 College of Materials Science and Engineering, Nanjing Tech University, Nanjing, 210009, China; 2 Institute of Nanotechnology, Karlsruhe Institute of Technology (KIT), Postfach 3640, 76021 Karlsruhe, Germany; 3 State Key Laboratory of Materials-Oriented Chemical Engineering, Nanjing Tech University, 210009 Nanjing, China

## Abstract

Metal oxychlorides are proved to be new cathode materials for chloride ion batteries. However, this kind of cathode materials is still in a very early stage of research and development. The obtained reversible capacity is low and the electrochemical reaction mechanism concerning chloride ion transfer is not clear. Herein, we report FeOCl/carbon composites prepared by mechanical milling of the as-prepared FeOCl with carbon nanotube, carbon black or graphene nanoplatelets as cathode materials for chloride ion batteries. The electrochemical performance of the FeOCl electrode is evidently improved by the incorporation of graphene into the cathode. FeOCl/graphene cathode shows a high reversible capacity of 184 mAh g^−1^ based on the phase transformation between FeOCl and FeO. Two stages of this phase transformation are observed for the FeOCl cathode. New insight into the reaction mechanism of chloride ion dissociation of FeOCl is investigated by DFT + U + D2 calculations.

The research in rechargeable batteries is mainly focused on the electrochemical systems based on cation transfer such as Li^+^, Na^+^, Mg^2+^ 1,2,3,4,5,6. In spite of the cation batteries, new kinds of batteries using the O_2_^−^, F^−^ or Cl^−^ anion for the electrochemical mass transfer have been reported recently^7^,^8^,^9^,^10^,^11^. Chloride ion battery shows high theoretical energy density, abundant material resources, high safety, and environmentally friendly features^10^,^11^, which fit the requirements for the development of new rechargeable battery systems. Moreover, Cl^−^ anion was found to have high mobility and high reversibility in the magnesium battery using metal chloride as cathode^12^, although it has higher ionic radius than Li^+^, Na^+^, Mg^2+^, O_2_^−^ or F^−^.

A key challenge of chloride ion batteries is to develop cathode materials which are stable in the electrolytes. Metal oxychlorides, which show higher stabilty and lower volumetric change than metal chlorides during cycling, have been proposed to be new cathode materials for chloride ion batteries^11^,^12^. The feasibility of the metal oxychloride cathodes has been proved in these first studys. However, the experimental discharge capacity is far from the corresponding theoretical discharge capacity. For instance, only about 30% of the theoretical discharge capacity (249.7 mAh g^−1^) of the FeOCl cathode was obtained at the fifth cycle^11^. FeOCl is a low-dimensional Mott insulator and has a high electrical resistivity of 10^7 ^Ω⋅cm at ambient conditions^13^,^14^. Moreover, a large volume contraction (−58.6%) or expansion (141.7%) can occur during the loss or return of chloride ion in FeOCl. The aforementioned low discharge capacity should be caused by the sluggish charge transfer and mass transfer of the FeOCl cathode during cycling. Therefore, the improvement on the conductivity and structural stability of the FeOCl cathode is necessary. Carbon material such as graphene^15^, carbon nanotube^16^, porous carbon^17^, carbon black^18^, activated carbon^19^ or their hybrid^20^ has been incorporated into the electrodes of rechargeable batteries. It could provide a conductive matrix and buffer volume change in the electrodes.

Herein, we report the FeOCl/carbon composite cathodes, which were prepared by mechanical milling the as-prepared pure FeOCl with graphene nanoplatelets (GN), carbon nanotube (CN) or carbon black (CB). The electrochemical performance and reaction mechanism of the FeOCl/carbon composite cathodes were investigated. Part of FeOCl was decomposed into FeCl_3_ and Fe_2_O_3_ when milling pure FeOCl or the FeOCl with carbon black, while this decomposition was restrained when the carbon material of graphene nanoplatelets or carbon nanotube was used during milling. The FeOCl/GN composite cathode shows a high reversible capacity of 184 mAh g^−1^ by the phase transformation between FeOCl and FeO. Two stages of this phase transformation were observed from the charge and discharge curves and the corresponding differential capacity plot. The DFT + U + D2 calculations have been performed to get an insight into the chloride ion dissociation process of FeOCl.

## Results and Discussion

**Figure 1:**
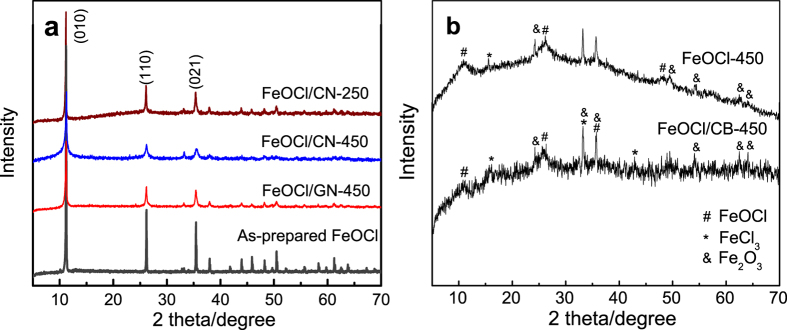
XRD patterns of the as-prepared FeOCl, the FeOCl/carbon composites and the mechanically milled FeOCl.

**Figure 2:**
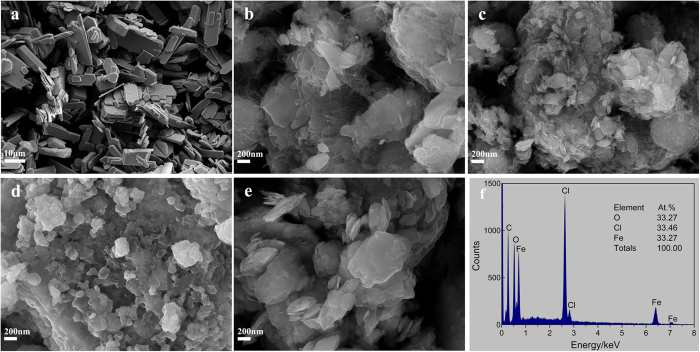
SEM images of the as-prepared FeOCl and FeOCl/carbon compsites: (**a**) FeOCl; (**b**) FeOCl/CN-250; (**c**) FeOCl/CN-450; (**d**) FeOCl/CB-450; and (**e**) FeOCl/GN-450. (**f**) the EDS pattern corresponding to the area in (**e**) of the FeOCl/GN-450 composite.

Figure 1 shows the XRD patterns of the as-prepared FeOCl, the FeOCl/carbon composites and the mechanically milled FeOCl. All reflections of the as-prepared FeOCl, FeOCl/CN and FeOCl/GN can be indexed and assigned to the orthorhombic layered FeOCl phase (PDF card no. 72–619) with three characteristic peaks corresponding to (010), (110) and (021) planes. The crystal structure of FeOCl was maintained during mechanical milling (MM) when GN or CN was used. MM results in the broadening and drastic decrease in the intensity of the diffraction peaks, refining the grains of the FeOCl phase. For instance, the FeOCl/CN composite milled at 450 rpm (FeOCl/CN-450) has an average grain size of 22 nm calculated according to the aforementioned three characteristic planes using the Scherrer equation, which is much smaller than 92 nm of the FeOCl/CN-250 sample. Furthermore, the large intensity of (010) plane indicates an orientation perpendicular to *b*-axis, facilitating the formation of a flake-like morphology during synthesis (Fig. 2a). The intensity ratio of the aforementioned three peaks is listed in Supplementary Table S1. MM reduced the intensity ratio; however, the FeOCl/CN and FeOCl/GN samples still shows high I_(010)_/I_(110)_ and I_(010)_/I_(021)_ values more than 2.5 and 2.2, respectively. Therefore, flake-like morphology was kept and nanosheets were formed after MM of FeOCl and CN or GN, as shown in Fig. 2b,c,e. The sample milled at a high speed possesses a smaller size. The EDS result for the FeOCl/GN sample in Fig. 2f demonstrates that the obtained FeOCl phase has a fine elemental composition. For the FeOCl/CB-450 sample, very fine grains of FeOCl phase were obtained after MM according to the weak and broad diffraction peaks (Fig. 1b). But the decomposition of FeOCl phase occurred because of the formation of α-Fe_2_O_3_ and FeCl_3_ phases. Similar decomposition occurred when the as-prepared pure FeOCl was milled at the same condition. Carbon black cannot limit this decomposition. Note that the FeOCl/CB-450 sample shows a very low I_(010)_/I_(110)_ or I_(010)_/I_(021)_ value of about 0.8 and accordingly a granular morphology. Maybe this is the caused by the decomposition of FeOCl, which prefers to have a stable structure with an orientation along (010) plane. CN or GN contributed to a dominant fracture along the (010) plane during milling and thus the decomposition of FeOCl was restrained.

**Figure 3:**
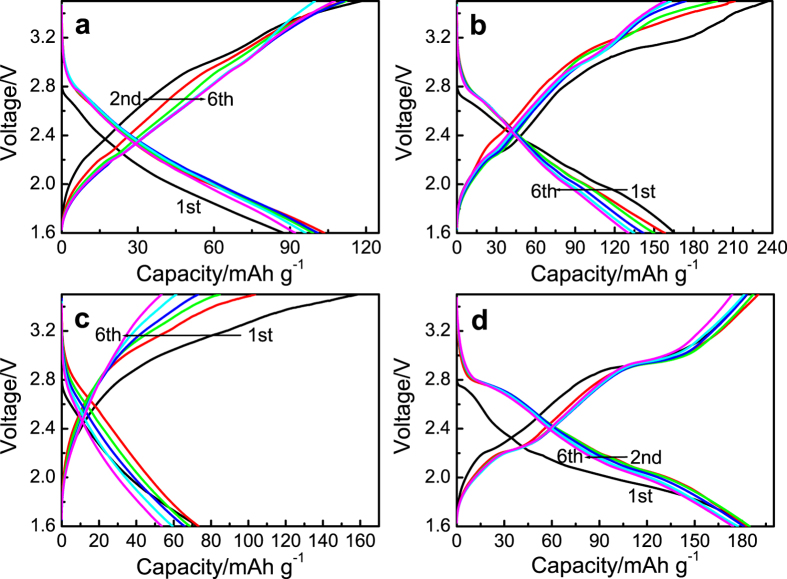
Discharge and charge curves (1st to 6th cycles, 10 mA g^−1^) of the FeOCl/Li electrode system using the electrolyte of 0.5M PP_14_Cl in PP_14_TFSI at 298 K: (**a**) FeOCl/CN-250 cathode; (**b**) FeOCl/CN-450 cathode; (**c**) FeOCl/CB-450 cathode; and (**d**) FeOCl/GN-450 cathode.

Figure 3 shows the discharge and charge curves of the FeOCl/Li electrode systems using different cathodes. The FeOCl/CN-250 cathode shows a discharge capacity of 103 mAh g^−1^, which is 41% of the theoretical capacity (249.7 mAh g^−1^), and a sloping discharge profile after activation (Fig. 3a). This low discharge capacity may be attributed to that part of FeOCl powders did not contribute to the electrochemical reaction. Some large FeOCl flakes without contact with CN could be found in Fig. 2b. Therefore, electrical contact would be blocked by these flakes and the utilization of the active material was limited. The FeOCl/CN sample prepared at a higher milling speed of 450 rpm possesses a better distribution between FeOCl and CN (Fig. 2c). Then the FeOCl/CN-450 cathode has a higher discharge capacity of 165 mAh g^−1^. Moreover, this cathode exhibits the discharge and charge profiles with different stages instead of only sloping profiles for the FeOCl/CN-250 cathode. For the FeOCl/CB-450 cathode, the sloping discharge and charge profiles are similar to those of the FeOCl/CB cathode prepared by a soft milling^11^. However, a maximum discharge capacity of only about 73 mAh g^−1^ and a low Coulombic efficiency of 46% were received at the first cycle. This may be caused by the decomposition of the FeOCl phase during the milling of FeOCl and CB (Fig. 1b), and thus the formation of Lewis acid FeCl_3_, which could be dissolved into the electrolyte by the formation of the soluble complex ion FeCl_4_^−^. As a result, the amount of the active material was decreased and the structural stability of the electrode would be weakened, leading to a depressed electrochemical performance.

Figure 3d shows the discharge and charge curves of the FeOCl/GN-450/Li electrode system. The FeOCl/GN-450 cathode shows a maximum discharge capacity of 184 mAh g^−1^ (73% of the theoretical discharge capacity) and a high Coulombic efficiency of 99% at the first cycle. Slight capacity decay was observed in the initial cycles. The electrochemical performance of the FeOCl electrode was evidently improved by the incorporation of graphene into the cathode by MM. Electrochemical impedance spectroscopy (EIS) measurement was carried out for the FeOCl/Li electrode systems using different FeOCl cathodes before cycling. The FeOCl cathode was served as the working electrode. The electrochemical processes of all the electrode systems show a controlling-step of a mixed rate-determining process containing charge transfer and chloride ion diffusion steps. For instance, the Nyquist plots (Supplementary Fig. S1a) of the FeOCl/CN-250 electrode consist of three parts, a semicircle at high frequency, followed by a further semicircle and a straight line at low frequency. This is consistent with the corresponding Bode-phase plots (Supplementary Fig. S1b) with three time constants. The former semicircle is considered to be the contact resistance, the later semicircle may be related to the charge transfer process, and the straight line should be associated with the Warburg impedance by chloride ion diffusion. It is evident that the FeOCl/CB electrode shows the highest electrochemical resistance, which was reduced when CN or GN was used. The FeOCl/GN-450 electrode possesses the best electrochemical kinetic performance, contributing to superior discharge and charge properties (Fig. 3d).

**Figure 4:**
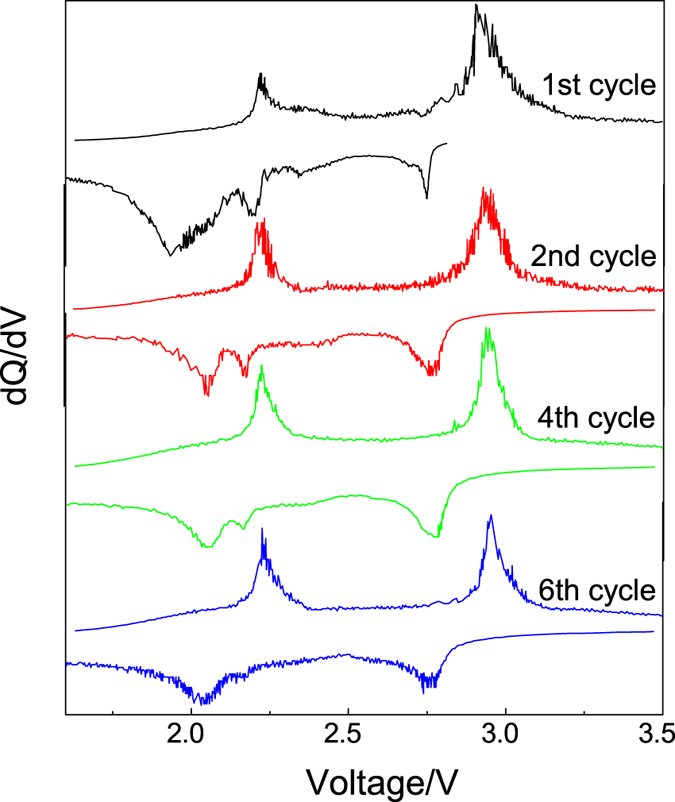
Differential capacity curves corresponding to the discharge and charge curves (Fig. 3d) of the FeOCl/GN-450/Li electrode system.

A more distinct two-step electrochemical process could be found in both the discharge and charge profiles of the FeOCl/GN-450 cathode as compared with the FeOCl/CN-450 cathode. The capacity was almost equally divided into two parts for the two steps, respectively. Figure 4 shows the corresponding differential capacity curves of the discharge and charge curves in Fig. 3d. Two pairs of the redox peaks could be observed in first six cycles. The first reduction peak appears at 2.77 V and the corresponding oxidation peak is located at 2.94 V. The other redox couple shows an oxidation peak at 2.22 V, followed by a small reduction peak at 2.16 V and a large reduction peak at 2.05 V. Generally, a single reduction peak should be formed. This might be due to the difference in the particle size and also the contact with graphene. Probably some FeOCl flakes have smaller particle size and better contact with graphene after MM, resulting in a higher discharge voltage plateau at 2.16 V. This small discharge plateau was weakened during cycling and almost disappeared at the 6th cycle. It means the distribution of the FeOCl/graphene regions tends to be uniform during cycling and thus a single reduction peak was formed. Similar phenomenon has been reported in the FeF_2_/carbon cathode for lithium ion battery^21^.

**Figure 5:**
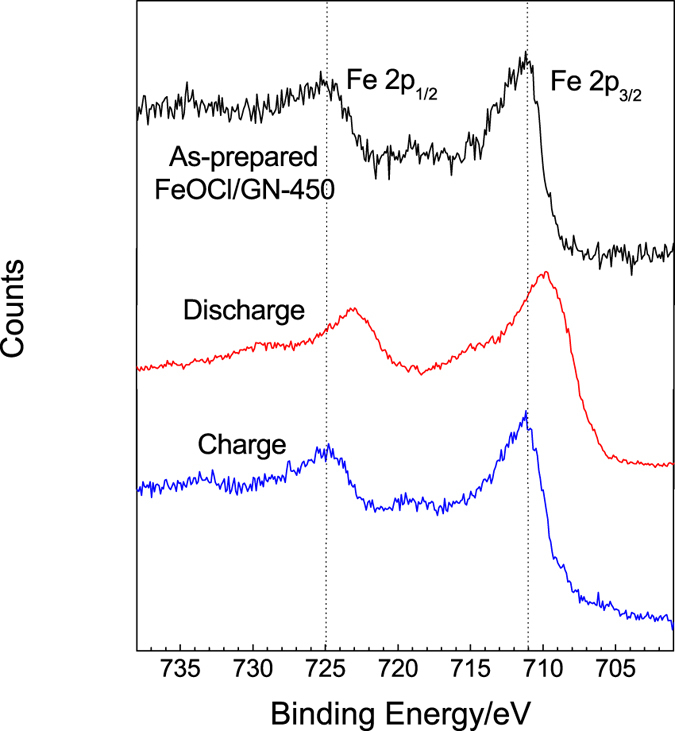
XPS region spectra of Fe 2p in the FeOCl/GN-450 cathode before and after cycling.

The reversible chloride ion transfer at metal oxychloride cathode (BiOCl or FeOCl) side has been proved by STEM/EDS/EELS in our previous work^11^. However, the product after discharge of FeOCl or VOCl was not detected by XRD^11^,^12^. This may be due to the formation of amorphous and/or nanosized particles. XPS was used to verify the structural evolution in the FeOCl/GN-450 electrode before and after cycling. Fe 2p_3/2_ and Fe 2P_1/2_ main peaks corresponding to the as-prepared pure FeOCl (Supplementary Fig. S2) and FeOCl/GN-450 (Fig. 5 and Supplementary Fig. S2) materials are located at 711.1 and 724.9 eV, respectively. When the FeOCl/GN-450/Li electrode system was fully discharged, the peaks of the FeOCl/GN-450 cathode at 709.6 and 723.0 eV were observed and can be assigned to FeO (Fe^2+^) phase^22^. For the charged electrode, the same Fe 2p signal as that of the as-prepared FeOCl/GN-450 material is received, indicating the formation of FeOCl by the reaction between FeO and Cl^−^ during charge and also the reversible reactions in the chloride ion battery.

**Figure 6:**
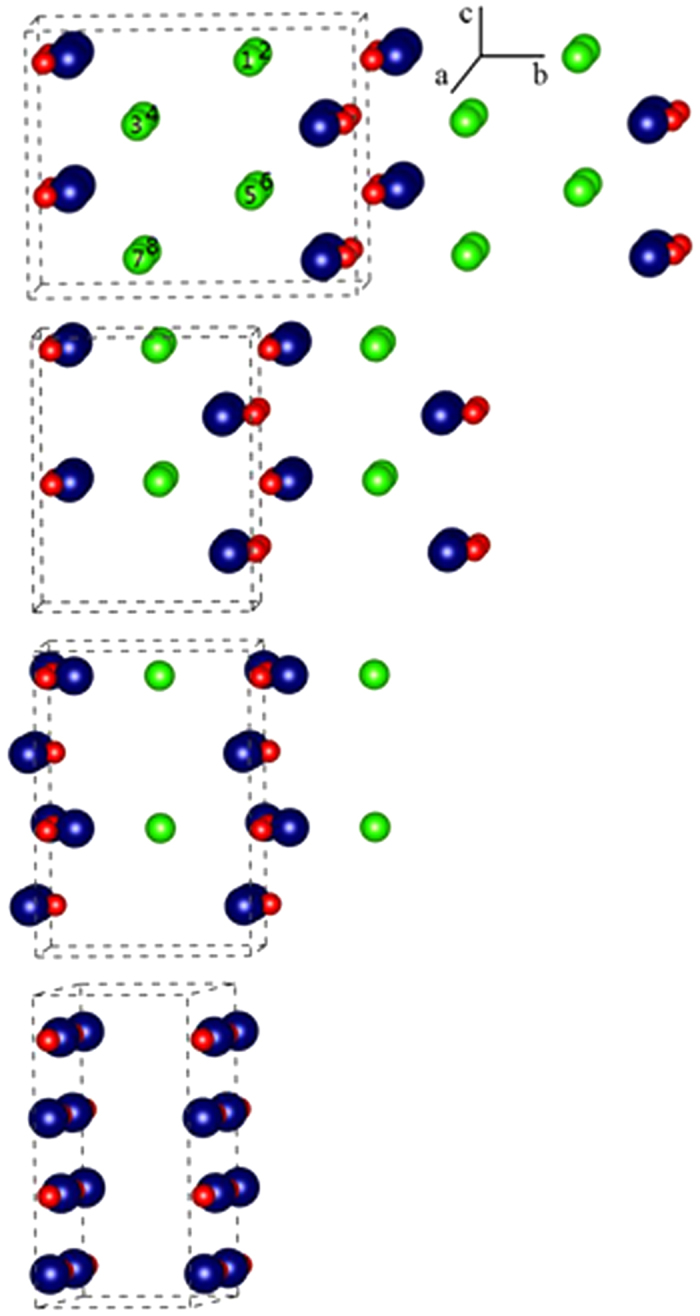
Optimized bulk structure of Fe_8_O_8_Cl_x_ (x = 0–8; Fe, bule; O, red; Cl, green) by DFT + U + D2. Top: Fe_8_O_8_Cl_8_; middle: Fe_8_O_8_Cl_4_ and Fe_8_O_8_Cl_2_; bottom: Fe_8_O_8_.

FeOCl/Li electrode system shows a one-electron electrochemical reaction. However, a two-step electrochemical process in both discharge and charge profiles (Fig. 3b,d) was obtained. Herein we employ DFT + U + D2 calculations to get an insight into the two-step chloride ion dissociation of FeOCl. The calculated lattice parameters of FeOCl have been improved in our previous work and agree well with the experimental data^12^. Each Fe^3+^ is coordinated with four oxygen ions and two chloride ions, and two neighboring ions are coupled anti-ferromagneticly to form a ground state without magnetism. A 2 × 1 × 2 super cell Fe_8_O_8_Cl_x_ (x = 0–8) was chosen to simulate the chloride ion dissociation process (Fig. 6).

We start with the removal of chloride ion 1 (Cl_1_) from Fe_8_O_8_Cl_8_ (perfect FeOCl crystal structure) in Fig. 6. The charges of two iron ions which connect to Cl_1_ are decreased by 0.17e^−^ according to the Bader charge analysis^23^,^24^,^25^. The reduced iron ions then move inward to the oxygen row, resulting in an increase in the lattice parameter a by about 0.2 Å, while the other two lattice parameters are barely changed. Another consequence is the interaction between the reduced iron ions and the neighboring chloride ions, Cl_5_ for example, is weakened. The calculated electrostatic potential energy based on equation (1) of Cl_5_ is −5.86 eV, which is much higher than −8.46, −8.76, −8.01, −7.39, −7.34 and −7.83 eV of Cl_2_, Cl_3_, Cl_4_, Cl_6_, Cl_7_ and Cl_8_, respectively. This indicates that Cl_5_ is less stable and thus the next dissociative chloride ion prefers to be Cl_5_. The chloride ions in the whole c-row (Cl_1_-Cl_5_) would dissociate subsequently (25% chloride ion dissociation). Then the neighboring Fe-row would be reduced and move towards to the adjacent oxygen row. The whole framework of FeOCl (Fe_8_O_8_Cl_6_), however, is almost unchanged; the lattice parameters b and c stay the same.

**Figure 7:**
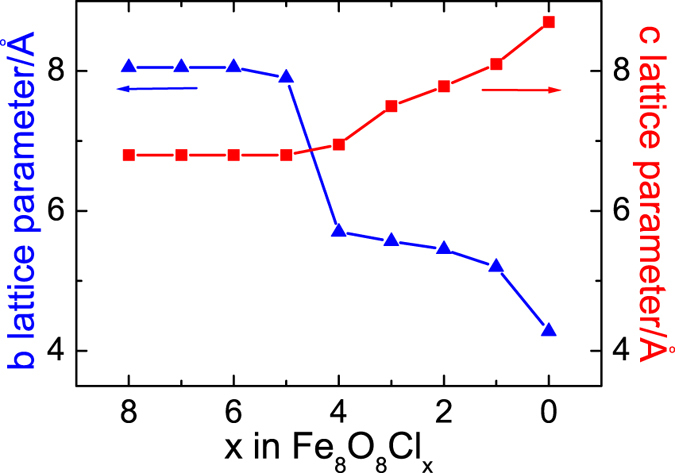
Changes in lattice parameters b and c upon removal of chloride ion from Fe_8_O_8_Cl_x_.

We then remove another c-row of chloride ions from our model system (Fe_8_O_8_Cl_4_, 50% chloride ion dissociation). The b-parameter drops dramatically by about 2 Å (Fig. 7); a and c parameters are slightly changed. -Fe-O- planes tend to be formed, since the reduced iron ions keep moving to oxygen rows. For the Fe_8_O_8_Cl_2_ (75% chloride ion dissociation) model, the transformation from FeOCl to FeO is almost finished. The b-parameter is decreased further and a,c-parameters are evidently increased to form Fe^2+^O^2+^ rows. In the end, a rock salt type FeO structure is obtained after fully dechlorination. Each iron ion connects to six oxygen ions, occupying an octahedral site. The optimized lattice parameter of FeO is 4.35 Å, which agrees well with the experimental data 4.30 Å^26^. This two-step chloride ion dissociation process is in accordance with the result of the electrochemical test.

## Conclusion

In summary, FeOCl/carbon composites have been prepared by mechanical milling the as-prepared FeOCl with carbon nanotube, carbon black or graphene nanoplatelets. The decomposition of part of FeOCl into Fe_2_O_3_ and FeCl_3_ during MM was restrained when carbon nanotube or graphene nanoplatelets was used. Graphene incorporated into the FeOCl cathode contributed to an improvement in the electrochemical kinetic performance and the obtained FeOCl/GN-450 cathode shows a high reversible capacity of 184 mAh g^−1^. This reversible reaction was proved to be according to the phase transformation between FeOCl and FeO by XPS. Moreover, the incorporation of carbon nanotube or graphene into the FeOCl cathode could result in a change in the discharge and charge profiles. The FeOCl/GN-450 cathode exhibits a distinct two-step discharge and charge process. New insight into the reaction mechanism of the chloride ion dissociation of FeOCl was investigated by DFT + U + D2 calculations, which show that one of the chlorine layers in FeOCl dissociates first, leading to a drastic decrease in the lattice parameter b of FeOCl. Then the loss of the other chlorine layer results in the formation of FeO.

## Methods

### Synthesis of Materials

The preparation of FeOCl using a chemical vapor transport method has been reported in our previous work^11^. FeOCl/carbon composite materials were fabricated by mechanical milling 1 g of the as-prepared FeOCl and 20 mass % graphene nanoplatelets aggregates (GN, Alfa Aesar), carbon nanotube (CN, Shanghai ANT) or carbon black (CB, Alfa Aesar) using a 50 ml agate vial with agate balls (10 and 6 mm in diameter) under an argon atmosphere. The ball to powder ratio was 40:1. The milling was performed in a planetary mill with a rotation speed of 250 or 450 rpm and the milling time was 10 h. The anhydrous ionic liquids of 1-Butyl-1-methylpiperidinium bis(trifluoromethylsulfonyl)imide (PP_14_TFSI, 99%, IoLiTech) and 1-Butyl-1-methylpiperidinium chloride (PP_14_Cl, 99%, IoLiTech) were all dried at 358 K for 72 h under vacuum.

### Structural analysis and electrochemical tests

X-ray Powder diffraction (XRD) was performed using a Rigaku SmartLab diffractometer with Cu-Kα radiation. The morphology and composition of the sample were characterized by a field-emission scanning electron microscopy (Ultra55 FE-SEM) incorporated with an energy-dispersive X-ray spectroscopy (EDS). X-ray photoelectron spectroscopy (XPS) measurements were conducted using a K-Alpha (Thermo Scientific) spectrometer with Al Ka radiation as the X-ray source. The vacuum in the analyzer chamber was kept at about 1 × 10^−9 ^mbar during the measurements. The C 1s line with a binding energy of 284.8 eV was used as a standard.

Electrochemical measurements were conducted using coin cells (CR2032) with lithium metal (Alfa Aesar) as anode. The cathode electrodes were fabricated by mixing as-synthesized material, PVDF, and carbon black in the mass ratio of 80:10:10. N-methyl-2-pyrrolidinone (NMP) was used as the solvent for PVDF to get homogeneous slurry, which was spread on a stainless steel (SS) foil and dried on hot plate at 373 K for 20 h. A mixture of 0.5 M PP_14_Cl in PP_14_TFSI was used as electrolyte. Celgard 2400 film was used as separator. Discharge and charge tests were carried out galvanostatically at 10 mA g^−1^ over a voltage range between 1.6 and 3.5 V by using Arbin BT2000 multi-channel battery testing system at 298 K. The specific capacities were calculated according to the corresponding active material of the cathode electrode. Electrochemical impedance spectroscopy (EIS) data were collected in the frequency range from 100 kHz to 10 mHz with an ac amplitude of 10 mV under a CHI660D electrochemical workstation.

### DFT calculations

We follow our previous work^27^, where we obtained the calculated lattice parameters (a = 3.851 Å, b = 8.050 Å and c = 3.298 Å) of FeOCl, which agree well with the experimental data. All of the spin-polarized density-functional theory (DFT) calculations were performed with the Vienna ab initio Simulation Package (VASP)^28^,^29^,^30^. The generalized gradient approximation with the functional described by Perdew *et al.* (GGA-PBE) was used for all calculations^31^. The projector-augmented wave (PAW) method is applied to describe the wavefunctions in the core regions^32^,^33^, while the valence wavefunctions are expanded as linear combination of plane-waves with a cutoff energy of 550 eV. The on-site Coulomb interactions were included only for strongly correlated Fe 3d electrons^34^,^35^, with an effective U value of 4.6^36^. DFT-D2 approaches were employed for the long-range dispersion corrections^37^,^38^,^39^.

Our unit cell for simulating the dissociation process is restrained to Fe_8_O_8_Cl_x_ (x = 0–8), the Brillouin zone was sampled with a (3 × 3 × 3) Monkhorst-Pack mesh of k-points^40^, which have been tested with respect to total energy of the system. In the optimization of the lattice constants, we always fix two lattice constants and optimize the third one; this was done iteratively until the changes were less than 0.02 Å.

Electrostatic potential energy is calculated upon the formula as follow,


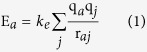


where *k*_*e*_ represents the Coulombic constant, *j* is the index for the point charges within a point charge field, which has been extended from an optimized unit cell consisting of 81000 point charges.

## Additional Information

**How to cite this article**: Zhao, X. *et al.* Carbon incorporation effects and reaction mechanism of FeOCl cathode materials for chloride ion batteries. *Sci. Rep.* **6**, 19448; doi: 10.1038/srep19448 (2016).

## Supplementary Material

Supplementary Information

## References

[b1] Wang C. *et al.* Fabrication and shell optimization of synergistic TiO_2_-MoO_3_ core-shell nanowire array anode for high energy and power density lithium-ion batteries. Adv. Funct. Mater. 25, 3524–3533 (2015).

[b2] Chen R. Y. *et al.* Disordered lithium-rich oxyfluoride as a stable host for enhanced Li^+^ intercalation storage. Adv. Energy Mater. 5, 1401814 (2015).

[b3] Yabuuchi N., Kubota K., Dahbi M. & Komaba S. Research development on sodium-ion batteries. Chem. Rev. 114, 11636–11682 (2014). 25390643 10.1021/cr500192f

[b4] Liu H. D., Xu J., Ma C. Z. & Meng Y. S. A new O3-type layered oxide cathode with high energy/power density for rechargeable Na batteries. Chem. Commun. 51, 4693–4696 (2015). 10.1039/c4cc09760b 25692397

[b5] Zhao-Karger Z. *et al.* Performance improvement of magnesium sulfur batteries with modified non-nucleophilic electrolytes. Adv. Energy Mater. 5, 1401155 (2015).

[b6] Okamoto S. *et al.* Intercalation and push-out process with spinel-to-rocksalt transition on Mg insertion into spinel oxides in magnesium batteries. Adv. Sci. 2, 1500072 (2015). 10.1002/advs.201500072 PMC5115418 27980965

[b7] Hibino M., Kimura T., Suga Y., Kudo T. & Mizuno N. Oxygen rocking aqueous batteries utilizing reversible topotactic oxygen insertion/extraction in iron-based perovskite oxides Ca_1–x_La_x_FeO_3-δ_. Sci. Rep. 2, 601 (2012). 22924108 10.1038/srep00601 PMC3426795

[b8] Rongeat C., Reddy M. A., Witter R. & Fichtner M. Solid electrolytes for fluoride ion batteries: ionic conductivity in polycrystalline tysonite-type fluorides. ACS Appl. Mater. Interfaces 6, 2103–2110 (2015). 24444763 10.1021/am4052188

[b9] Rongeat C., Reddy M. A., Diemant T., Behm R. J. & Fichtner M. Development of new anode composite materials for fluoride ion batteries. J. Mater. Chem. A 2, 20861–20872 (2014).

[b10] Zhao X. Y., Ren S., Bruns M. & Fichtner M. Chloride ion battery: a new member in the rechargeable battery family. J. Power Sources 245, 706–711 (2014).

[b11] Zhao X. Y., Zhao-Karger Z., Wang D. & Fichtner M. Metal oxychlorides as cathode materials for chloride ion batteries. Angew. Chem., Int. Ed. 52, 13621–13624 (2013). 10.1002/anie.201307314 24346944

[b12] Zhao X. Y. *et al.* Vanadium oxychloride/magnesium electrode systems for chloride ion batteries. ACS Appl. Mater. Interfaces 6, 22430–22435 (2014). 25419861 10.1021/am5064266

[b13] Kim S. H., Kang J. K., Hwang S. & Kim H. A theoretical study on the electronic structures of MOCl (M=Ti, V and Fe) and their relationship with physical properties. Bull. Korean Chem. Soc. 16, 299–304 (1995).

[b14] Bykov M. *et al.* High-pressure behavior of FeOCl. Phys.Rev. B 88, 014110 (2013).

[b15] Raccichini R., Varzi A., Passerini S. & Scrosati B. The role of graphene for electrochemical energy storage. Nature Mater. 14, 271–279 (2015). 25532074 10.1038/nmat4170

[b16] Li R. Z. *et al.* Carbon-stabilized high-capacity ferroferric oxide nanorod array for flexible solid-State alkaline battery-supercapacitor hybrid device with high environmental suitability, Adv. Funct. Mater. 25, 5384–5394 (2015).

[b17] Jung D. S. *et al.* Hierarchical porous carbon by ultrasonic spray pyrolysis yields stable cycling in lithium–sulfur battery. Nano Lett. 14, 4418–4425 (2014). 25007002 10.1021/nl501383g

[b18] Park C. M. & Sohn H. J. Black phosphorus and its composite for lithium rechargeable batteries. Adv. Mater. 19, 2465–2468 (2007).

[b19] Elazari R., Salitra G., Garsuch A., Panchenko A. & Aurbach D. Sulfur-impregnated activated carbon fiber cloth as a binder-Free Cathode for rechargeable Li-S batteries. Adv. Mater. 23, 5641–5644 (2011). 22052740 10.1002/adma.201103274

[b20] Tang C. *et al.* Nitrogen-Doped Aligned Carbon Nanotube/graphene sandwiches: Facile catalytic growth on bifunctional natural catalysts and their applications as scaffolds for high-Rate lithium-sulfur batteries. Adv. Mater. 26, 6100–6105 (2014). 24862890 10.1002/adma.201401243

[b21] Reddy M. A. *et al.* CF_x_ Derived carbon–FeF_2_ nanocomposites for reversible lithium storage. Adv. Energy Mater. 3, 308–313 (2013).

[b22] Grosvenor A. P., Kobe B. A., Biesinger M. C. & McIntyre N. S. Investigation of multiplet splitting of Fe 2p XPS spectra and bonding in iron compounds. Surf. Interface Anal. 36, 1564–1574 (2004).

[b23] Tang W., Sanville E. & Henkelman G. A grid-based bader analysis algorithm without lattice bias. J. Phys. Condens. Matter. 21, 084204 (2009). 21817356 10.1088/0953-8984/21/8/084204

[b24] Sanville E., Kenny S. D., Smith R. & Henkelman G. Improved grid-based algorithm for bader charge allocation J. Comput. Chem. 28, 899–908 (2007). 17238168 10.1002/jcc.20575

[b25] Henkelman G., Arnaldsson A. & Jónsson H. A fast and robust algorithm for bader decomposition of charge density. Comput. Mater. Sci. 36, 354–360 (2006).

[b26] Yamamoto A. Modulated structure of wustite (Fe_1–*x*_O) (three-dimensional modulation). Acta Cryst. B 38, 1451–1456 (1982).

[b27] Zhao X. Y. *et al.* Magnesium anode for chloride ion batteries. ACS Appl. Mater. Interfaces 6, 10997–11000 (2014). 24999978 10.1021/am503079e

[b28] Kresse G. & Hafner J. Ab Initio Molecular dynamics for open-shell transition metals. Phys.Rev. B 48, 13115–13118 (1993). 10.1103/physrevb.48.13115 10007687

[b29] Kresse G. & Furthmüller J. Efficiency of ab-initio total energy calculations for metals and semiconductors. Comput. Mater. Sci. 6, 15–50 (1996).

[b30] Kresse G. & Furthmüller J. Efficient iterative schemes for ab initio total-energy calculations using a plane-wave basis set. Phys. Rev. B 54, 11169–11186 (1996). 10.1103/physrevb.54.11169 9984901

[b31] Perdew J. P., Burke K. & Ernzerhof M. Generalized gradient approximation made simple. Phys. Rev. Lett. 77, 3865–3868 (1996). 10062328 10.1103/PhysRevLett.77.3865

[b32] Kresse G. & Joubert D. From ultrasoft pseudopotentials to the projector augmented-wave method. Phys. Rev. B 59, 1758–1775 (1999).

[b33] Blöchl P. E. Projector augmented-wave method. Phys. Rev. B 50, 17953–17979 (1994). 10.1103/physrevb.50.17953 9976227

[b34] Rohrbach A., Hafner J. & Kresse G. Electronic correlation effects in transition-metal sulfides. J. Phys.: Condens. Matter 15, 979–996 (2003).

[b35] Dudarev S. L., Botton G. A., Savrasov S. Y., Humphreys C. J. & Sutton A. Electron-energy-loss spectra and the structural stability of nickel oxide: an LSDA+U study. Phys. Rev. B 57, 1505–1509 (1998).

[b36] Liao P. & Carter E. A. Ab initio DFT + U predictions of tensile properties of iron oxides. J. Mater. Chem. 20, 6703–6719 (2010).

[b37] Grimme S., Antony J., Ehrlich S. & Krieg H. A Consistent and accurate ab initio parametrization of density functional dispersion correction (DFT-D) for the 94 Elements H-Pu. J. Chem. Phys. 132, 154104 (2010). 20423165 10.1063/1.3382344

[b38] Grimme S. Semiempirical GGA-Type Density functional constructed with a long-range dispersion correction. J. Comput. Chem. 27, 1787–1799 (2006). 16955487 10.1002/jcc.20495

[b39] Bučko T., Hafner J., Lebègue S. & Ángyán J. G. Improved description of the structure of molecular and layered crystals: ab initio DFT calculations with van der Waals corrections. J. Phys. Chem. A 114, 11814–11824 (2010). 20923175 10.1021/jp106469x

[b40] Monkhorst H. J. & Pack J. D. Special points for brillouin-zone integrations. Phys. Rev. B 13, 5188–5192 (1976).

