# Supplementary material for: Carbon incorporation effects and reaction mechanism of FeOCl cathode materials for chloride ion batteries
Source: Sci Rep. 2016 Jan 18;6:19448. doi: 10.1038/srep19448 (PMC4726103; doi:10.1038/srep19448)
Supplement: Supplementary Information [file srep19448-s1.pdf]

Supplementary Information

Carbon incorporation effects and reaction mechanism of FeOCl cathode materials for chloride ion batteries

Xiangyu Zhao,[1,]* Qiang Li,[2] Tingting Yu,[1] Meng Yang,[1] Karin Fink[2] and Xiaodong Shen[1,3]

[1]College of Materials Science and Engineering, Nanjing Tech University, 210009 Nanjing, China
[2]Institute of Nanotechnology, Karlsruhe Institute of Technology (KIT), 76021 Karlsruhe, Germany
[3]State Key Laboratory of Materials-Oriented Chemical Engineering, Nanjing Tech University, 210009 Nanjing, China

Tabel S1. The intensity ratio of the diffraction peaks corresponding to (010), (110), and (021) planes of FeOCl

**Samples**	$\mathbf{I_{(010)}/I_{(021)}}$	$\mathbf{I_{(110)}/I_{(021)}}$	$\mathbf{I_{(010)}/I_{(110)}}$
**As-prepared FeOCl**	3.71	1.20	2.58
**FeOCl/CN-250**	2.80	1.14	2.46
**FeOCl/CN-450**	2.55	1.12	2.27
**FeOCl/GN-450**	2.52	1.10	2.29
**FeOCl/CB-450**	0.81	0.92	0.88
**FeOCl-450**	0.87	0.98	0.89

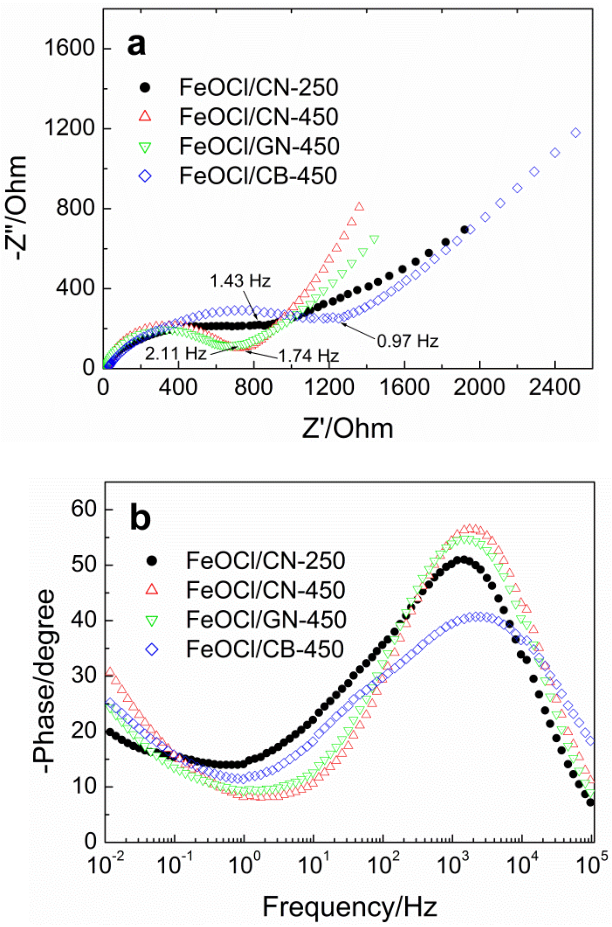

Figure S1. EIS patterns of the FeOCl/Li electrode systems using different FeOCl cathodes before cycling: a) Nyquist plots and b) Bode-phase plots.

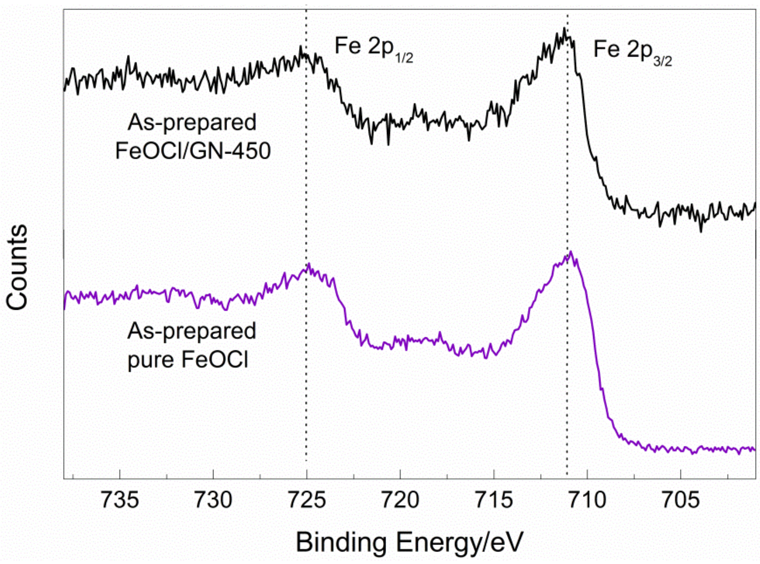

Figure S2. XPS region spectra of Fe 2p in the as-prepared pure FeOCl and FeOCl/GN-450 materials.
